# Artificial Light-at-Night Exposure and Overweight and Obesity across GDP Levels among Chinese Children and Adolescents

**DOI:** 10.3390/nu15040939

**Published:** 2023-02-13

**Authors:** Jiajia Dang, Di Shi, Xi Li, Ning Ma, Yunfei Liu, Panliang Zhong, Xiaojin Yan, Jingshu Zhang, Patrick W. C. Lau, Yanhui Dong, Yi Song, Jun Ma

**Affiliations:** 1 Institute of Child and Adolescent Health, School of Public Health, Peking University, Beijing 100191, China; 2 National Health Commission Key Laboratory of Reproductive Health, Peking University, Beijing 100191, China; 3 State Key Laboratory of Information Engineering in Surveying, Mapping and Remote Sensing, Wuhan University, Wuhan 430000, China; 4 Collaborative Innovation Centre of Geospatial Technology, Wuhan University, Wuhan 430000, China; 5 Department of Sport, Physical Education and Health, Hong Kong Baptist University, Kowloon Tong, Hong Kong 999077, China

**Keywords:** artificial light-at-night, overweight, obesity, children, adolescents

## Abstract

Background: Evidence in adults suggests that exposure to artificial light-at-night (ALAN) leads to obesity. However, little is known about whether this effect exists in children and adolescents. We aimed to investigate whether ALAN exposure was associated with overweight and obesity in school-aged children and adolescents and whether this association varied with socioeconomic status. Methods: Data on the height and weight of 129,500 children and adolescents aged 10–18 years from 72 cities were extracted from the 2014 Chinese National Survey on Students’ Constitution and Health (CNSSCH). The ALAN area percentage and average ALAN intensity were calculated using the Visible/Infrared Imager/Radiometer Suite. The subjects were separated into three categories based on the cities’ gross domestic product per capita (GDPPC). A mixed-effect logistic regression model and generalized additive model (GAM) were utilized to evaluate the association between ALAN exposure and overweight and obesity in children and adolescents stratified by municipal GDPPC. Results: Both ALAN area (OR = 1.194, 95% CI: 1.175–1.212) and ALAN intensity (OR = 1.019, 95% CI: 1.017–1.020) were positively associated with overweight and obesity in children and adolescents, and the associations remained robust after adjusting for covariates. ORs for overweight and obesity and ALAN area decreased as GDPPC level increased (first tertile: OR = 1.457, 95% CI: 1.335–1.590; second tertile: OR = 1.350, 95% CI: 1.245–1.464; third tertile: OR = 1.100, 95% CI: 1.081–1.119). Similar results were observed for ALAN intensity. In the GAM models, thresholds existed in almost all these spline trends, indicating that ALAN might have a nonlinear association with overweight and obesity. Conclusions: ALAN contributed to the development of overweight and obesity in children and adolescents and this effect differed with GDPPC. Future longitudinal studies should confirm the causal relationship between ALAN and obesity. Moreover, reducing unnecessary exposure to artificial light at night may have beneficial implications for controlling childhood and adolescent obesity, particularly in low-income areas.

## 1. Introduction

Overweight and obesity in childhood and adolescence have become a major public health issue globally, as they can induce type 2 diabetes, metabolic abnormalities, cardiovascular disease, and even cancer in adulthood, resulting in severe medical burdens [1,2]. China, as the country with the largest number of obese children and adolescents in the world [3], has an alarming obesity epidemic. According to national prevalence estimates for 2015–2019 [4], 11.1% of school-aged children (7–17 years) and adolescents were overweight and 7.9% were obese, increasing from 1.1% and 0.2% in 1985 [5]. It was also projected that the prevalence of overweight and obesity among school-aged students in China might reach 31.8% by 2030, and the number of overweight and obese children and adolescents might reach 58.92 million [6]. Traditional risk factors for overweight and obesity in children and adolescents, such as insufficient physical activity, excessive dietary intake, and sedentary behavior, have been explored and their relevance confirmed [7]; however, the causes of obesity cannot be fully explained by self-health behaviors. In addition, researches have shown that individuals, especially children, face obstacles in maintaining a healthy lifestyle, and the outcomes are commonly unsatisfactory [8,9]. Given these findings, it is crucial to explore easily implementable environmental intervention targets for obesity in children and adolescents, such as regarding the influence of artificial light-at-night (ALAN), which has become increasingly acknowledged [10,11].

ALAN is one of the environmental endocrine disruptors that have changed most obviously in recent decades. In 2016, ALAN covered 25% of the world’s land area and more than 80% of people were exposed to it [12], while China ranked 12th for the proportion of land area and 15th for population exposure among G20 countries [13]. On the one hand, the widespread application of ALAN has brought tremendous social benefits [14], profoundly altering our lifestyles and greatly enhancing the night sky’s luminosity [15]. On the other hand, ALAN has also significantly disrupted natural light patterns and has progressively been recognized as a new source of pollution that contributes to human health hazards, such as circadian rhythm disorder, breast cancer, and obesity [16,17]. Current research has focused on the association between ALAN and obesity and metabolic disorders in adults [18,19,20], especially shift workers exposed to higher levels of ALAN, but little is known for children and adolescents [21,22]. Considering that children and adolescents are in a critical stage of growth and development that is different from adults, high exposure to ALAN may possibly be an important reason for childhood obesity and metabolic abnormalities [23,24]. In addition, there may be potential health inequities in regions with different levels of economic development [25]. Gross domestic product per capita (GDPPC) is a statistically important indicator reflecting public health and socioeconomic status [26]. Given the close relationship between ALAN and socioeconomic development, it is necessary to explore the association between ALAN and overweight and obesity in children and adolescents using representative epidemiological investigations from diverse socioeconomic circumstances.

In the present study, we investigated whether ALAN exposure is associated with overweight and obesity in children and adolescents and whether this association varies with GDPPC level using nationally representative data from the 2014 Chinese National Survey of Students’ Constitution and Health (CNSSCH). We hypothesized that exposure to ALAN would be positively associated with overweight and obesity in children and adolescents, and the association would differ when stratified by GDPPC.

## 2. Materials and Methods

### 2.1. Study Design and Participants

**Figure 1:**
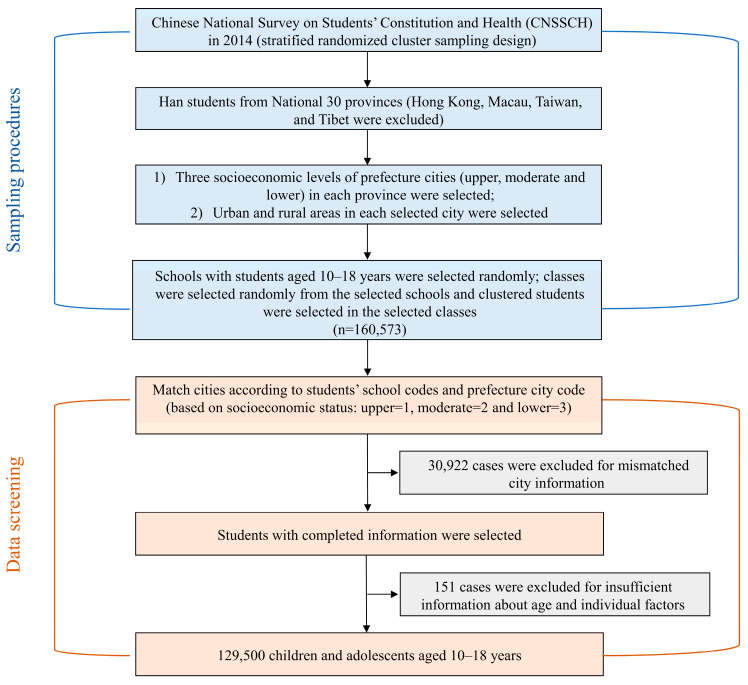
Flowchart for data from 2014 CNSSCH.

We investigated the nutritional status of school-aged children and adolescents using data from the 2014 Chinese National Survey of Students’ Constitution and Health (CNSSCH), the largest national cross-sectional survey of students, which was conducted in 30 provinces, autonomous regions, and municipalities in China (it was not conducted in Macau, Hong Kong, Taiwan, and Tibet). The CNSSCH sampling procedures were employed following [27] and are shown in Figure 1. We used stratified cluster random sampling. Subjects were sampled from randomly selected classes for each grade level from randomly chosen schools. Each province was classified into two area groups (urban and rural areas) according to residential regions. Equal numbers were obtained through the sampling for each of the residency statuses (urban and rural) and for both sexes. 

This study included 129,500 Han children and adolescents aged 10–18 years with complete city and basic characteristics information. The project was approved by the Medical Research Ethics Committee of the Peking University Health Science Center (IRB00001052-19095). Informed consent was obtained from both children and their parents. Participants’ information was anonymized and de-identified prior to analysis to protect their privacy.

### 2.2. Assessment of ALAN Exposure

Launched on 28 October 2011, the Visible/Infrared Imager/Radiometer Suite (VIIRS) collected high-quality images at night at a spatial resolution of 750 m in the 500–900 nm day/night bands (DNBs) [28]. Monthly, cloud-free, global calibrated mosaics compiled using night light VIIRS images [29] are now available from the National Oceanic and Atmospheric Administration’s National Geoscience Data Center [30]. We collected and processed data to obtain cloud-free coverage from which the yearly VIIRS brightness data could be constructed, with the predicted variables being ALAN brightness and lit area for each city. Employing the Defense Meteorological Program (DMSP) Operational Line-Scan System (OLS) as the main global ALAN data source, most studies on ALAN exposure use lit area instead of the ALAN brightness calibration value. Although both variables were found to be highly correlated with the research variables, the relationship between lit area and mean brightness has been found to be nonlinear [31]. Thus, two quantitative indicators of outdoor ALAN at 1:30 AM for each city were extracted and estimated in this study: (a) the proportion of area covered by ALAN (ALAN area) and (b) the average ALAN intensity (lx) (ALAN intensity). In addition, we also used digital number values (DN values) to represent the distributions of ALAN and research cities in China in 2014. The DN value is the pixel brightness value for remote sensing image and is equal to the calculated total DN value/number of grids.

### 2.3. Outcome Measurement

Height (in cm) and weight (in kg) were measured by trained technicians using a standardized procedure. Participants were asked to wear light clothing and stand on the measuring instrument without shoes to ensure the accuracy of the measurement. In order to control for measurement errors, the researchers repeated the measurements of height and weight for 3 percent of the randomly selected participants. Body mass index (BMI) was then calculated as the weight (kg) divided by the square of the height (m). Age- and sex-specific BMI Z-scores were also calculated. We analyzed overweight (>+1 SD for BMI Z-score) and obesity (>+2 SD for BMI Z-score) using the age- and sex-specific BMI (kg/m^2^) standards defined by the WHO [32]. Overweight (OW), obesity (OB), and overweight and obesity (OW and OB) were used as the different BMI levels. The reference group was considered as normal weight and thinness (≤+1 SD for BMI Z-score). 

### 2.4. Questionnaire Survey and Procedures

A self-reported questionnaire was used to collect lifestyle information, including daily sleep duration, whether the participant ate breakfast, whether the participant drank milk, whether the participant ate eggs, time spent watching TV, total screen time, work time, running exercise, sports preferences, and physical activity time. Information about screen time, sleep duration, and physical activity was collected based on the reported daily time spent on these activities. Breakfast, milk, and egg intake was grouped into four levels (never, 1~2 days/week, 3~5 days/week, everyday). Prior to the questionnaire survey, the researchers introduced the content of the survey and provided enough time to ensure that children and adolescents had a good understanding of the survey. Researchers were available to provide appropriate assistance and guidance as needed. Epidata version 3.1 (Odense, Denmark) was used to input the answers for all questionnaires. All survey items were checked for completeness and accuracy by field surveyors and program directors, and an overall survey response of 99%, moderate internal consistency, and Cronbach’s α reliability coefficient of 0.68 were achieved [27]. In addition, the survey collected demographic information on age, sex, and residency status. 

We generated several contextual covariates, including sociodemographic information and individual lifestyle factors. We also collected information on the municipal development indicators measured by the GDPPC and resident populations from the National Bureau of Statistics of China [33]. The GDPPC was categorized into three levels according to the tertiles of the cities: first tertile (USD 2559–6318 per year); second tertile (USD 6319–11,298 per year); and third tertile (USD 11,299–32,306 per year). Additionally, we divided the GDPPC into three strata (there were no low-income cities in this study) based on the international universal cutoff points for country affluence established by the World Bank in 2013 as a sensitivity analysis: lower middle income—USD 1036–4085 per year; upper middle income—USD 4086–12,616 per year; and high income—>USD 12,616 per year.

### 2.5. Statistical Analysis

Little’s Missing Completely At Random (MCAR) test was used to test whether samples were randomly missing. Quantitative variables were analyzed as medians (interquartile ranges) according to the normality of distribution, and qualitative variables were analyzed as numbers (percentages). The Kruskal–Wallis test and chi-squared test were used to compare difference between sub-groups. Scatter plots were used to validate correlations between ALAN indicators and overweight and obesity for each city in 2014. Mixed-effect logistic regression models were applied, and adjusted odds ratios (ORs) and 95% confidence intervals (CIs) were calculated for associations between ALAN indicators and overweight and obesity stratified by various GDPPC levels. Generalized additive models (GAMs) were further used to calculate the nonlinear fitting curve of the coefficients of the ALAN indicators and overweight and obesity to quantify differences between different GDPPC levels. To confirm the robustness of the different GDPPC stratifications, we performed a mixed-effect logistic regression analysis based on the World Bank classification method published in 2013. The effects of sociodemographic characteristics (age, sex, and residence), individual life factors (sleep duration, exercise time, breakfast, egg and milk intake, and screen time), and municipal population information, as well as the clustering effects of schools, were adjusted for all model analyses. All statistical analyses were performed with ArcGIS version 10.6 (Environmental Systems Research Institute, Inc., RedLands, California, USA) and R version 4.1.0 (R Development Core Team, Vienna, Austria ). A two-tailed *p* value of < 0.05 was considered statistically significant. 

## 3. Results

### 3.1. Characteristics of Participants

**Figure 2:**
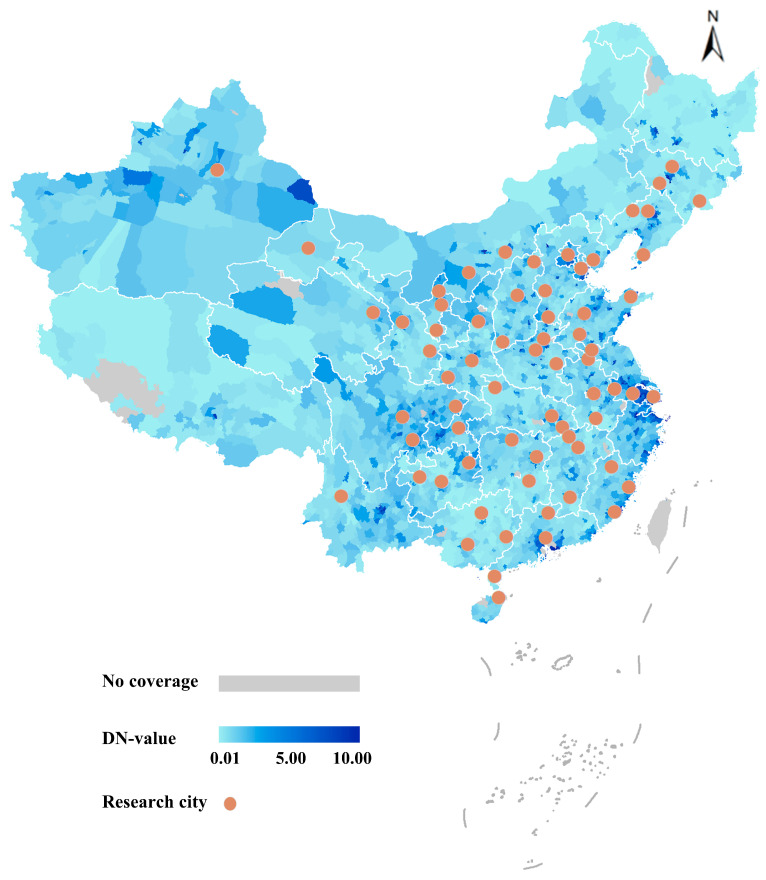
Distributions of ALAN and research cities in China in 2014. DN value: digital number value. The DN value is equal to the calculated total DN value/number of grids. Data were obtained from the Chinese Research Data Services (CNRDS) Platform.

A total of 30,922 cases were excluded due to mismatched city information and 151 cases were excluded due to insufficient information on age and individual factors, but the loss was found to be random after employing Little’s MCAR test (*p* > 0.05). We included 129,500 participants with complete records for basic characteristics and questionnaire information in the final analyses (Figure 1). The final dataset contained 72 cities from 29 provinces (Hong Kong, Macau, Taiwan, Tibet, and Heilongjiang were not included). Figure 2 shows the distribution map for ALAN (DN value) for the 72 study cities in China in 2014. The characteristics of the children and adolescents are summarized in Table 1. The prevalence of overweight was 12.5% and the prevalence of obesity was 6.3%. Participants with high levels of GDPPC were associated with greater height, weight, BMI, prevalence of overweight and obesity, ALAN area, and ALAN intensity than those with low levels of GDPPC (*p* < 0.001).

### 3.2. Distribution of ALAN Exposure and Overweight and Obesity

**Figure 3:**
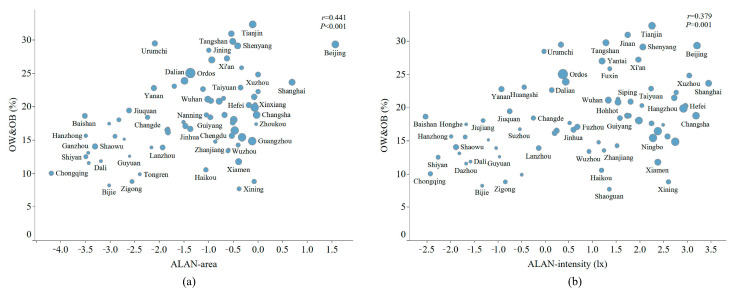
Distribution of the prevalence of overweight and obesity based on ALAN exposure. (**a**) The prevalence of overweight and obesity based on ALAN area; (**b**) the prevalence of overweight and obesity based on ALAN intensity. Log-transformation was undertaken for the ALAN indictors. Each dot represents a city, and the size of the dot represents the level of GDP per capita at the city level. r represents the correlation index, and the P value is for the correlation index. OW: overweight; OB: obesity; ALAN: artificial light-at-night.

Figure 3 shows the distributions of the prevalence of overweight and obesity and ALAN exposure. After adjusting for municipal GDPPC, the correlation between the prevalence of overweight and obesity and ALAN indicators remained significant (ALAN area: r = 0.441, *p* < 0.001; ALAN intensity: r = 0.379, *p* = 0.001). Overweight and obesity were more strongly associated with ALAN area (Figure 3a) than ALAN intensity (Figure 3b). The overall prevalence of overweight and obesity and ALAN exposure in each city are shown in Figure S1 in the Supplementary Materials.

### 3.3. Associations between ALAN and Overweight and Obesity Stratified by GDPPC

**Figure 4:**
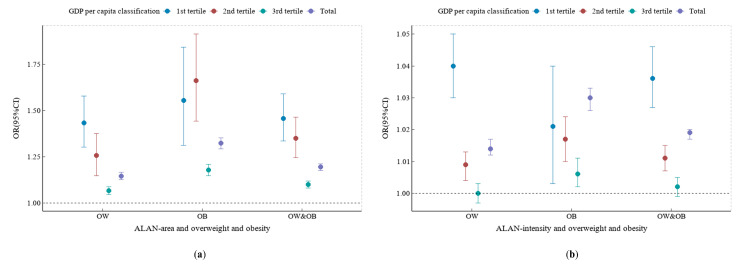
Adjusted ORs for ALAN indicators and overweight and obesity across GDP per capita tertiles. (**a**) ALAN area and overweight and obesity; (**b**) ALAN intensity and overweight and obesity. Modes were adjusted for the fixed effects of age, sex, residence, sleep duration, exercise time, screen time, breakfast, egg and milk intake, and city-level population, as well as the clustering effect of school. Tertiles of regional gross domestic product (USD): first tertile = (2559–6318); second tertile = (6319–11298); third tertile = (11299–32306). ALAN: artificial light-at-night; OW: overweight; OB: obesity.

Figure 4 summarizes the results for the association between ALAN indicators and overweight and obesity stratified by GDPPC. Relatively, both ALAN area (OR = 1.194, 95% CI: 1.175–1.212) and ALAN intensity (OR = 1.019, 95% CI: 1.017–1.020) were positively associated with overweight and obesity, and the associations remained robust after adjusting for covariates. OR values for overweight and obesity and ALAN exposure decreased with the increase in GDPPC levels, except for the association between ALAN area and OB. For example, the OR values for ALAN area and OW and OB decreased gradually with the increase in GDPPC level (first tertile: OR = 1.457, 95% CI: 1.335–1.590; second tertile: OR = 1.350, 95% CI: 1.245–1.464; third tertile: OR = 1.100, 95% CI: 1.081–1.119). Stronger associations were observed for OB than OW for the same GDPPC classification. The results for the associations between ALAN indicators and overweight and obesity in accordance with the World Bank 2013 GDPPC classification were consistent with those for the tertile classifications of the GDPPC (see Figure S2 in the Supplementary Materials). Specific OR values are shown in Tables S1 and S2 in the Supplementary Materials.

**Figure 5:**
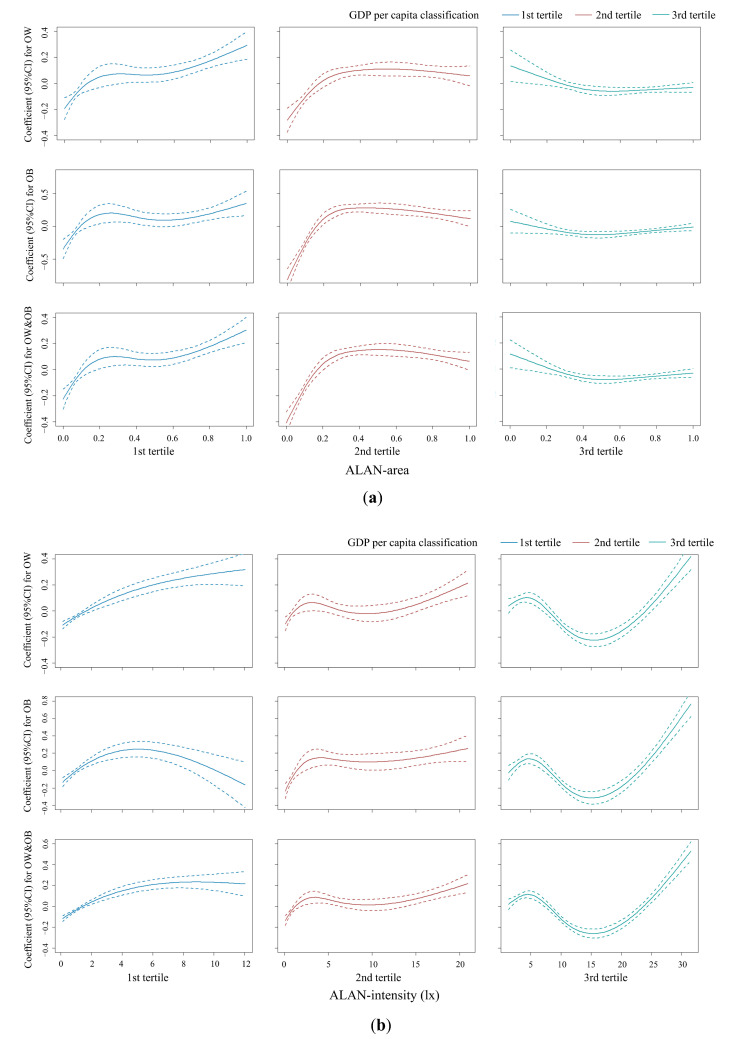
Generalized additive models for ALAN indicators and overweight and obesity across GDP per capita tertiles. (**a**) ALAN area and overweight and obesity; (**b**) ALAN intensity and overweight and obesity. Modes were adjusted for the fixed effects of age, sex, residence, sleep duration, exercise time, screen time, breakfast, egg and milk intake, and city-level population, as well as the clustering effect of school. Dashed lines represent 95% CI bands. CI: confidence interval; ALAN: artificial light-at-night; OW: overweight; OB: obesity. Dotted red, blue, and green lines indicate 95% CIs.

In the GAM models, the associations between ALAN exposure and overweight and obesity showed similar trends at different BMI levels (OW, OB, and OW and OB) (Figure 5). However, there were significant differences in these associations between GDPPC levels. Thresholds existed for almost all these spline trends. The associations between ALAN and overweight and obesity had a pattern of increasing first and then decreasing as the GDPPC level increased. The strength of the relationship between ALAN area and overweight and obesity was greater in low GDPPC areas, while ALAN intensity was similar in different GDPPC areas and higher in the highest group.

## 4. Discussion

To the best of our knowledge, this was the first study to evaluate the association between outdoor ALAN exposure and overweight and obesity in nationwide representative school-aged children and adolescents. We discovered that higher ALAN exposure was positively associated with increased risks of overweight and obesity, and the association differed between GDPPC levels after controlling for the influence of other confounding factors, such as GDPPC, eating habits, screen time, sleep duration, and physical activity. The results may provide evidence to help policymakers reduce the health burden of overweight and obesity in children and adolescents.

Our findings add new evidence regarding the effects of outdoor ALAN exposure on overweight and obesity in children and adolescents. The results were consistent with the findings of previous adult studies. A global cross-sectional study found that outdoor ALAN has become a positive predictor of overweight and obesity in both sexes in over 80 countries around the world, and the effects of ALAN on overweight were comparable to those of junk food [34]. Using a follow-up period of more than 10 years, another prospective investigation revealed a substantial association between higher outdoor ALAN exposure and increased risk of obesity in adults [35]. In addition, evidence of an association between outdoor ALAN and obesity has been identified from the study of Old Order Amish individuals who have not been exposed to common sources of indoor ALAN, such as TVs, computers, and electrical lights [36], suggesting that the effects of outdoor ALAN are independent of indoor ALAN exposure [37]. Therefore, we believe that outdoor ALAN is an important environmental exposure factor rather than a surrogate indicator of other urban environmental exposures. In addition, previous studies stated that the association between ALAN and obesity is mainly mediated by sleep duration or sleep characteristics but, after controlling for these factors, the association between ALAN and obesity remained unchanged [20,38]. Our findings support the importance of ALAN, as an environmental endocrine disruptor, for human health.

Stronger associations between ALAN area and overweight and obesity were found in underdeveloped regions, while associations between ALAN intensity and overweight and obesity were stronger in highly developed regions. There was evidence that GDPPC was associated with an increase in childhood overweight and obesity [25,39]. Although both GDPPC growth and ALAN exposure have effects on overweight and obesity in children and there may be collinearity between the two, this study found that ALAN exposure was still positively correlated with overweight and obesity at different GDPPC levels, demonstrating that the effect of ALAN on overweight and obesity was independent. There are numerous underdeveloped districts in China, and ALAN exposure might play a crucial role in the development of overweight and obesity in recent years. Understanding the mechanisms underlying the diverse effects of ALAN at varying GDPPC levels is critical for targeting obesity interventions. It is possible that the strong impacts of ALAN on overweight and obesity in children and adolescents in underdeveloped areas were due to their greater exposure to environments deficient in effective curtain shelters, convenient and diverse food supplies, and more accessible activity venues. The effects of outdoor ALAN on overweight and obesity might be offset by other influencing factors, such as the use of blackout curtains in highly developed areas. However, in areas with the highest levels of economic development, ALAN intensity was associated with higher rates of overweight and obesity, probably due to the effects of increased use of electronics on the circadian and endocrine rhythms.

Existing evidence indicates that ALAN might influence body weight and metabolism in the following ways. Continuous exposure to ALAN affects the suprachiasmatic nucleus (SCN, the primary circadian oscillator in mammals) [37], resulting in a free-running circadian system (i.e., one that is not controlled by the rhythm of the 24 h day–night cycle) [37]. Furthermore, ALAN alters the circadian clock genes in the SCN and peripheral tissues [40,41]. The circadian system is involved in maintaining energy homeostasis [42]. Rhythmic genes that play a specific role in regulating nutrient metabolism can also be disrupted when ALAN disrupts the circadian rhythm system [43], as well as the expression of hormones [44,45]. These endocrine and metabolic changes can contribute to the development of overweight and obesity. A recent study showed that optogenetically induced sharp wave ripples (SPW-RS), generated during slow-wave sleep in the hippocampus, regulate endocrine metabolism [46]. The positive associations between ALAN exposure and the risk of overweight and obesity may be explained by ALAN-induced physiological changes, which in turn lead to behavioral changes, such as changes in food intake and sleep time, shifting from the active stage to the rest stage [18,38]. Research on children and adolescents in China suggested that outdoor ALAN may have adverse effects on sleep duration [47]. Evidence suggests that individuals who consume more food after 8:00 PM tend to have a higher BMI and shorter sleep duration, which increases their risks for obesity, weight gain, and higher body fat composition over time [48].

Given that ALAN is a single potential environmental risk factor, it does not affect the human body in the manner of direct toxicity or physical energy like other environmental risk factors, such as chemical poisons or radiation [49]. Therefore, it is difficult to explain the biological mechanism of ALAN itself. However, many existing studies have shown that circadian rhythm disturbance and melatonin inhibition are possible ways that ALAN may affect human health [50]. This study suggests that future work should measure the circadian rhythm period and metabolic hormone changes in children and adolescents to explore the direct or indirect pathways and mechanisms linking ALAN and obesity.

We utilized nationwide data to evaluate the association between ALAN and overweight and obesity in children and adolescents. However, this research had limitations. Firstly, satellite images of ALAN might not be able to reflect individual artificial light pollution exposure levels, which might be insufficient to influence the circadian rhythm. In addition, outdoor ALAN measured via satellite was not validated for individual ALAN exposure, since indoor ALAN from TVs, computers, and smartphones could also affect individuals [18,38]. A study of children in eight Chinese cities found that use of TV and other media had a negative impact on sleep quality [51]. Furthermore, indoor ALAN also had negative impact on cardiometabolic health in young adults [52]. Given these findings, ALAN exposure from indoor screen-based electronic devices is an issue worth considering. However, the period required for the collection of individual indoor ALAN exposure is long and the process is limited by equipment, so it is difficult to simultaneously measure individual indoor ALAN exposure for a large sample population. Secondly, we only adjusted for a few covariables in this study, and there might be residual confounders, such as mutations, genetics, and other sleep-related variables, including sleep–wake patterns and social jetlag, that are associated with both ALAN exposure and obesity [53]. Individuals are not only exposed to ALAN but also experience asynchronous circadian rhythm behavior, making it difficult to determine the effect of ALAN alone [37]. It has been proposed that social jetlag might be an important influence and a potential factor to explore in the relationship between ALAN and obesity. Thirdly, we only used the GDPPC, which is a relatively simple indicator of economic development, and a comprehensive indicator should be considered in the future. Finally, the positive association between outdoor ALAN and obesity found in our study might not directly indicate a causal relationship.

## 5. Conclusions

In summary, our analyses of a large population of Chinese school-aged children and adolescents suggested that ALAN might contribute to the development of overweight and obesity and that this effect differs with GDPPC level. We emphasize that ALAN should and likely will be a focus for children and adolescents with overweight and obesity. Reducing unnecessary ALAN exposure in early childhood, particularly in economically disadvantaged areas, and establishing a regular rest schedule may strengthen metabolic regulation in the future, with important implications for reducing childhood overweight and obesity.

## Figures and Tables

**Table 1:** The characteristics of the study participants.

Characteristics	Total (N = 129,500)	GDP per Capita (USD)			*p*
		First Tertile	Second Tertile	Third Tertile	
Age (year)	14.5(4.5)	14.5(4.5)	14.4 (4.4)	14.5 (4.4)	0.125
Sex					0.957
Boys	64,767 (50.0)	21,226 (50.1)	21,291 (50.0)	22,250 (50.0)	
Girls	64,733 (50.0)	21,179 (49.9)	21,267 (50.0)	22,287 (50.0)	
Residence					<0.001
Urban	63,844 (49.3)	21,222 (50.0)	21,256 (49.9)	21,360 (48.0)	
Rural	65,668 (50.7)	21,183 (50.0)	21,302 (50.1)	23,177 (52.0)	
Height (cm)	159.1 (15.4)	157.5 (15.2)	159.0 (15.4)	161.0 (15.0)	<0.001
Weight (kg)	49.8 (15.9)	48.1 (15.1)	49.5 (15.9)	51.7 (16.3)	<0.001
BMI (kg/m^2^)	19.3 (4.2)	19.0 (4.0)	19.3 (4.2)	19.7 (4.4)	<0.001
Overweight (%)	16,249 (12.5)	4922 (11.6)	5750 (13.5)	6980 (15.7)	<0.001
Obesity (%)	8160 (6.3)	1420 (3.3)	2097 (4.9)	3012 (6.8)	<0.001
ALAN area	0.4 (0.6)	0.1 (0.5)	0.3 (0.5)	0.6 (0.5)	<0.001
ALAN intensity (lx)	3.3 (8.9)	0.4 (3.7)	1.2 (8.9)	7.8 (12.0)	<0.001
Sleep duration (h)	7.5 (2.0)	7.5 (2.0)	7.5 (2.0)	7.5 (2.0)	<0.001
Screen time (h)	1.3 (1.3)	1.3 (1.5)	1.3 (1.3)	1.3 (1.5)	<0.001
Physical activity (min)	45.0 (15.0)	45.0 (15.0)	45.0 (15.0)	45.0 (45.0)	<0.001
Daily breakfast (%)					<0.001
never	2187 (1.7)	873 (2.1)	588 (1.4)	726 (1.6)	
1–2 days/week	8004 (6.2)	3293 (7.8)	2335 (5.5)	2375 (5.3)	
3–5 days/week	22,868 (17.7)	8691 (20.5)	7420 (17.4)	6755 (15.2)	
everyday	96,453 (74.5)	29,548 (69.7)	32,215 (75.7)	34,681 (77.9)	
Daily milk (%)					<0.001
never	8192 (6.3)	3263 (7.7)	2582 (6.1)	2347 (5.3)	
1–2 days/week	77,813 (60.1)	29,392 (69.3)	24,959 (58.6)	23,454 (52.7)	
3–5 days/week	36,399 (28.1)	8315 (19.6)	12,796 (30.1)	15,284 (34.3)	
everyday	7108 (5.5)	1435 (3.4)	2221 (5.2)	3452 (7.8)	
Daily eggs (%)					<0.001
never	13,541 (10.5)	5108 (12.0)	4830 (11.3)	3602 (8.1)	
1–2 days/week	66,184 (51.1)	24,108 (56.9)	21,373 (50.2)	20,698 (46.5)	
3–5 days/week	33,927 (26.2)	9738 (23.0)	11,080 (26.0)	13,105 (29.4)	
everyday	15,860 (12.2)	3451 (8.1)	5275 (12.4)	7132 (16.0)	

Note: data are expressed as the median (interquartile range) or frequency value (percentage, %); GDP: gross domestic product; BMI, body mass index; ALAN: artificial light-at-night.

## Data Availability

The data supporting the conclusions of this article obtained from the corresponding author upon request.

## Supplementary Materials

The following supporting information can be downloaded at: https://www.mdpi.com/article/10.3390/nu15040939/s1, Figure S1. Distribution of prevalence of overweight and obesity and ALAN exposure in China. (a) Prevalence of overweight and obesity on the basis of ALAN area; (b) prevalence of overweight and obesity on the basis of ALAN intensity. Figure S2. Adjusted ORs for ALAN indictors and overweight and obesity across GDPPC strata. (a) ALAN area and overweight and obesity; (b) ALAN intensity and overweight and obesity. Table S1. Associations between ALAN indictors and overweight and obesity across GDPPC tertiles. Table S2. Associations between ALAN indictors and overweight and obesity across GDPPC strata.
