# Supplementary material for: Artificial Light-at-Night Exposure and Overweight and Obesity across GDP Levels among Chinese Children and Adolescents
Source: Nutrients. 2023 Feb 13;15(4):939. doi: 10.3390/nu15040939 (PMC9961462; doi:10.3390/nu15040939)
Supplement: Supplementary file 1 [file nutrients-15-00939-s001.zip › nutrients-2071956-supplementary.pdf]

Artificial light-at-night exposure and overweight and obesity across GDP levels among Chinese children and adolescents

Figure S1. Distribution of prevalence of overweight and obesity and ALAN exposure in China. (**a**) Prevalence of overweight and obesity on the basis of ALAN-area; (**b**) prevalence of overweight and obesity on the basis of ALAN-intensity.

Figure S2. Adjusted ORs between ALAN indictors and overweight and obesity across strata of GDP per capita. (**a**) ALAN-area and overweight and obesity; (**b**) ALAN-intensity and overweight and obesity.

Table S1. Associations between ALAN indictors and overweight and obesity across tertiles of GDP per capita.

Table S2. Associations between ALAN indictors and overweight and obesity across strata of GDP per capita.

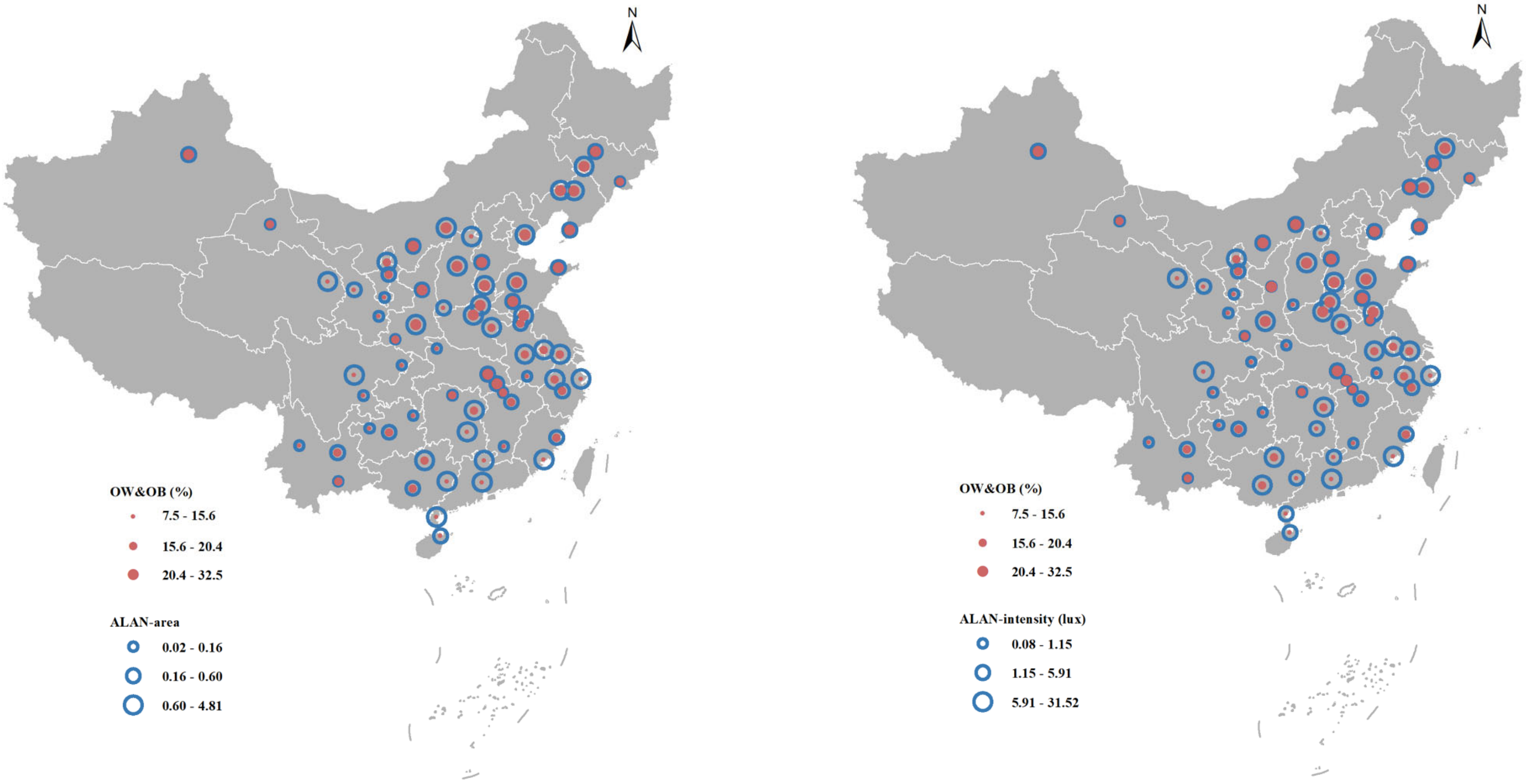

(**a**) (**b**)

Figure S1. Distribution of prevalence of overweight and obesity and ALAN exposure in China. (**a**) Prevalence of overweight and obesity on the basis of ALAN-area; (**b**) prevalence of overweight and obesity on the basis of ALAN-intensity; OW: overweight; OB: obesity; ALAN: artificial light-at-night.

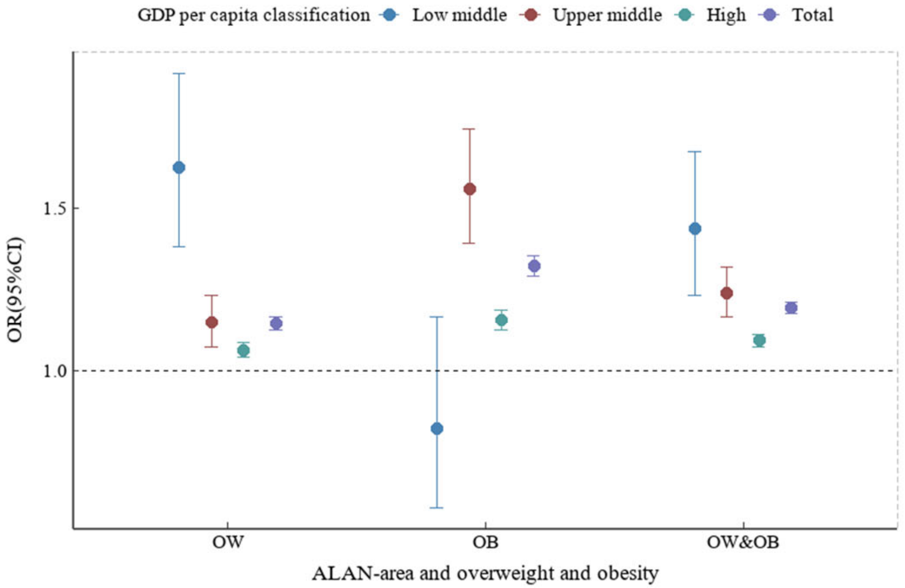

(**a**)

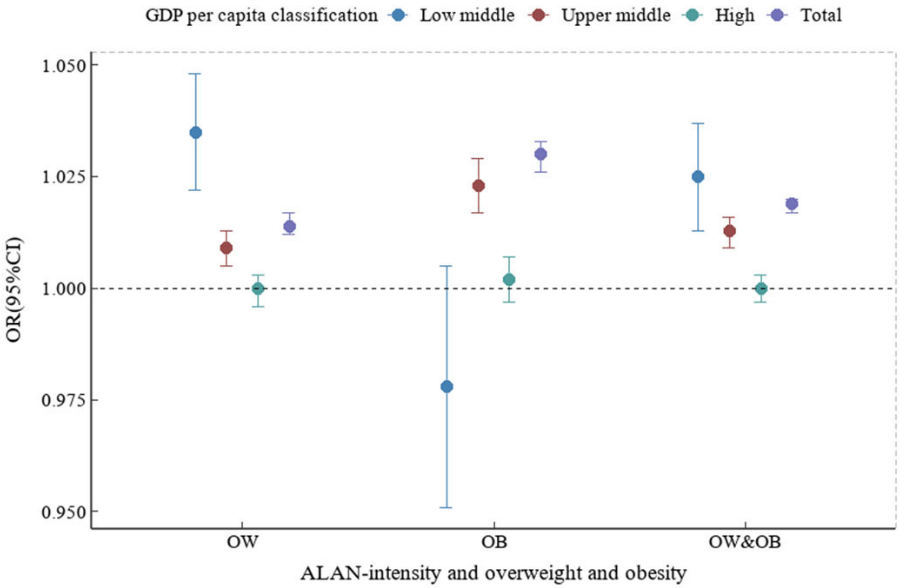

(**b**)

Figure S2. Adjusted ORs between ALAN indictors and overweight and obesity for different strata of GDP per capita. (**a**) ALAN-area and overweight and obesity; (**b**) ALAN-intensity and overweight and obesity. Modes were adjusted for the fixed effect of age, sex, residence, sleep duration, exercise time, screen time, breakfast, eggs and milk intake, city-level population, and the clustering effect of school. Classification of Regional gross domestic product per capita (US$): low middle income= (1,036–4,085); upper middle income = (4,086–12,616); High income >12,616. ALAN: artificial light-at-night; OW: overweight; OB: obesity.

Table S1. Associations between ALAN indictors and overweight and obesity for different tertiles of GDP per capita.

Body weight measures	GDP per capita [a]	ALAN-area		ALAN-intensity	
		OR	95% CI	OR	95% CI
OW [b]	Total	1.144	(1.124,1.165)	1.014	(1.012,1.017)
	1st tertile	1.432	(1.301,1.577)	1.040	(1.030,1.050)
	2nd tertile	1.255	(1.146,1.375)	1.009	(1.004,1.013)
	3rd tertile	1.066	(1.045,1.088)	1.000	(0.997,1.003)
OB [b]	Total	1.322	(1.292,1.352)	1.030	(1.026,1.033)
	1st tertile	1.554	(1.311,1.842)	1.021	(1.003,1.040)
	2nd tertile	1.661	(1.442,1.913)	1.017	(1.010,1.024)
	3rd tertile	1.177	(1.147,1.208)	1.006	(1.002,1.011)
OW&OB [b]	Total	1.194	(1.175,1.212)	1.019	(1.017,1.020)
	1st tertile	1.457	(1.335,1.590)	1.036	(1.027,1.046)
	2nd tertile	1.350	(1.245,1.464)	1.011	(1.007,1.015)
	3rd tertile	1.100	(1.081,1.119)	1.002	(0.999,1.005)

Note: [a] Tertiles of Regional gross domestic product per capita (US$): 1st tertile = (2,559–6,318); 2nd tertile = (6,319–11,298); 3rd tertile = (11,299–32,306). [b] Adjusted for the fixed effect of age, sex, residence, sleep duration, exercise time, screen time, breakfast, eggs and milk intake, city-level population, and the clustering effect of school. OR: odds ratio; CI, confidence interval; ALAN: artificial light-at-night.

Table S2. Associations between ALAN indictors and overweight and obesity for different strata of GDP per capita according to the World Bank classification in 2013.

Body weight measures	GDP per capita [a]	ALAN-area		ALAN-intensity	
		OR	95% CI	OR	95% CI
OW [b]	Total	1.144	(1.124,1.165)	1.014	(1.012,1.017)
	Low middle income	1.625	(1.380,1.913)	1.035	(1.022,1.048)
	Upper middle income	1.149	(1.071,1.232)	1.009	(1.005,1.013)
	High income	1.062	(1.040,1.085)	1.000	(0.996,1.003)
OB [b]	Total	1.322	(1.292,1.352)	1.030	(1.026,1.033)
	Low middle income	0.822	(0.579,1.167)	0.978	(0.951,1.005)
	Upper middle income	1.559	(1.393,1.744)	1.023	(1.017,1.029)
	High income	1.155	(1.123,1.187)	1.002	(0.997,1.007)
OW&OB [b]	Total	1.194	(1.175,1.212)	1.019	(1.017,1.020)
	Low middle income	1.437	(1.232,1.674)	1.025	(1.013,1.037)
	Upper middle income	1.239	(1.164,1.320)	1.013	(1.009,1.016)
	High income	1.091	(1.071,1.111)	1.000	(0.997,1.003)

Note: [a] Classification of Regional gross domestic product per capita (US$): low middle income= (1,036–4,085); upper middle income = (4,086–12,616); High income >12,616. [b] Adjusted for the fixed effect of age, sex, residence, sleep duration, exercise time, screen time, breakfast, eggs and milk intake, city-level population, and the clustering effect of school. OR: odds ratio; CI, confidence interval; ALAN: artificial light-at-night.
